# Compounds from* Cynomorium songaricum* with Estrogenic and Androgenic Activities Suppress the Oestrogen/Androgen-Induced BPH Process

**DOI:** 10.1155/2017/6438013

**Published:** 2017-05-15

**Authors:** Xueni Wang, Rui Tao, Jing Yang, Lin Miao, Yu Wang, Jose Edouard Munyangaju, Nuttapong Wichai, Hong Wang, Yan Zhu, Erwei Liu, Yanxu Chang, Xiumei Gao

**Affiliations:** ^1^Tianjin State Key Laboratory of Modern Chinese Medicine, 312 Anshanxi Road, Nankai District, Tianjin 300193, China; ^2^Institute of Traditional Chinese Medicine, Tianjin University of Traditional Chinese Medicine, 312 Anshanxi Road, Nankai District, Tianjin 300193, China; ^3^Key Laboratory of Pharmacology of Traditional Chinese Medical Formulae, Ministry of Education, Tianjin University of Traditional Chinese Medicine, Tianjin 300193, China

## Abstract

**Objective:**

To investigate the phytoestrogenic and phytoandrogenic activities of compounds isolated from CS and uncover the role of CS in prevention of oestrogen/androgen-induced BPH.

**Methods:**

Cells were treated with CS compounds, and immunofluorescence assay was performed to detect the nuclear translocation of ER*α* or AR in MCF-7 or LNCaP cells; luciferase reporter assay was performed to detect ERs or AR transcriptional activity in HeLa or AD293 cells; MTT assay was performed to detect the cell proliferation of MCF-7 or LNCaP cells. Oestrogen/androgen-induced BPH model was established in rat and the anti-BPH, anti-estrogenic, and anti-androgenic activities of CS in vivo were further investigated.

**Results:**

The nuclear translocation of ER*α* was stimulated by nine CS compounds, three of which also stimulated AR translocation. The transcriptional activities of ER*α* and ER*β* were induced by five compounds, within which only ECG induced AR transcriptional activity as well. Besides, ECG stimulated the proliferation of both MCF-7 cells and LNCaP cells. CS extract suppressed oestrogen/androgen-induced BPH progress in vivo by downregulation of E2 and T level in serum and alteration of the expressions of ER*α*, ER*β*, and AR in the prostate.

**Conclusion:**

Our data demonstrates that compounds from CS exhibit phytoestrogenic and phytoandrogenic activities, which may contribute to inhibiting the oestrogen/androgen-induced BPH development.

## 1. Introduction

**Figure 1:**
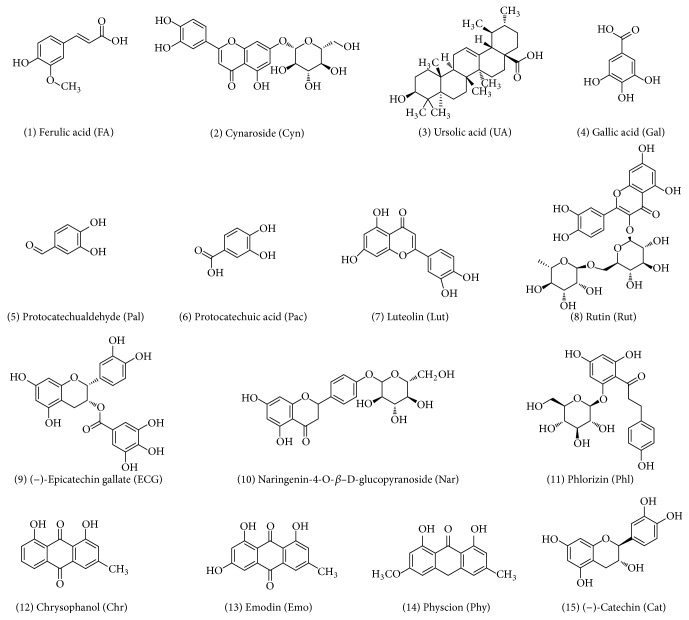
Chemical structures of compounds isolated from* Cynomorium songaricum* (CS).

*Cynomorium songaricum* (CS) is a traditional Chinese medicine (TCM) that has been practically used for treatment of hormone deficient diseases, including sexual dysfunction, infertility, deficient kidney function, and prostatic diseases for hundreds of years [1–3]. A variety of compounds isolated from CS have been identified and classified as pentacyclic triterpene, flavonoid, flavonoid glycoside, and anthraquinones (Figure 1) [4, 5]. However, the mechanism by which CS and its compounds regulate estrogen and/or androgen signaling remains unclear.

It has been reported that natural compounds may exhibit phytoestrogenic activity through multiways such as induction of estrogen receptor (ER) nuclear translocation, stimulation of ER transcriptional activity, and promotion of estrogen-dependent cell proliferation, thereby behaving like estradiol [6, 7]. Phytoestrogens participate in estrogen-related signaling as either ER antagonists or agonists and thus are called selective estrogen receptor modulators (SERMs). It has been reported that phytoestrogens have protective effects against breast cancer, prostate cancer, and cardiovascular diseases [8]. Comparison to that of phytoestrogens identification of phytoandrogenic activity from natural compounds is arising recently [9]. Concerning the potential treatment for androgen-regulated diseases like benign prostatic hyperplasia and prostate cancer, several natural compounds have been recognized as agonists or antagonists against androgens [10–15], and display the tissue-selective activation of androgenic signaling [16], which are so called selective androgen receptor modulators (SARMs) by competitively binding to androgen receptor (AR).

Benign prostatic hyperplasia (BPH) is an age-related common disease in older men [17], in which both androgen and estrogen signaling [18, 19] are involved via their specific receptors. Studies from different groups have showed that ER*α* (one subtype of ER) and AR are overexpressed in BPH tissues and knocking down either of them significantly blocks BPH progression in vivo [20, 21], indicating their positive roles for BPH development [22, 23]. On the other hand, ER*β* (the other subtype of ER) exhibits antiproliferation activity that suppresses BPH development as a negative factor in the prostate [24]. Therefore, downregulation of ER*α* and AR or upregulation of ER*β* could become effective ways and hopeful targets that contribute to BPH therapeutics.

CS is an important anti-BPH herbal medicine in China [25], while the mechanism is still uncovered well. Here we first analyzed the phytoestrogenic and phytoandrogenic activities of compounds isolated from CS and then investigated whether the anti-BPH effect of CS in oestradiol/testosterone (1 : 100)-induced BPH was due to interference with androgen and/or estrogen signaling.

## 2. Materials and Methods

### 2.1. Extract and Compounds

*Cynomorium songaricum *extract was prepared by desiccation after reflux with 70% ethanol. Ferulic acid (FA), cynaroside (Cyn), ursolic acid (UA), gallic acid (Gal), protocatechualdehyde (Pal), protocatechuic acid (Pac), luteolin (Lut), rutin (Rut), epicatechin gallate (ECG), naringenin-4-O-b-D-glucopyranoside (Nar), phlorizin (Phl), chrysophanol (Chr), emodin (Emo), physcion (Phy), catechin (Cat) were isolated from the extract as previously described [26]. Chemical structures of compounds were shown in Figure 1.

### 2.2. Reagents and Plasmids

Dihydrotestosterone (DHT) was purchased from Solarbio (Beijing China). 17*β*-estradiol (E2) and Tamoxifen (Tam) were obtained from Sigma-Aldrich. Lipofectamine® 2000 Transfection Reagent was from life technologies (USA). Hoechst 33342 was purchased from Cell Signaling Technology (USA). RPMI 1640 was purchased from Sigma (USA). Fetal Bovine Serum was from Hyclone (New Zealand). Charcoal stripped FBS was from Biological Industries (USA). Dual-Luciferase® Reporter Assay was from Promega (USA).

Mammalian ER*α*, ER*β*, and AR expression vectors and the estrogen response element (ERE) and the androgen response element luciferase reporter plasmids pTk-ERE-luc and pTk-ARE-luc were gifts from Dr. ZhuYan (Tufts Medical Center, Boston, USA) and Dr. J. Zhang (Nankai University, Tianjin, China) separately. pTk-Renilla was purchased from Promega.

### 2.3. Cell Culture

MCF-7 cells were obtained from professor Zhang (Peking University, Beijing). Hela cells were purchased from Institute of Biochemistry and Cell Biology (Shanghai, China). MCF-7 cells and HeLa cells were maintained in DMEM supplemented with 10%, 100 units/mL penicillin, and 100 *μ*g/*μ*L streptomycin. LNCaP and AD293 cells were obtained from professor Zhang Ju (Nankai University, Tianjin) and maintained in RPMI 1640 supplemented with 10% (v/v) fetal bovine serum (FBS) (HyClone, New Zealand), 100 units/mL penicillin, and 100 *μ*g/*μ*L streptomycin. In experiments requiring androgen or estrogen or compound stimulation, cells were cultured in phenol red-free medium supplemented with 10% charcoal stripped FBS.

### 2.4. Immunocytofluorescence Imaging

MCF-7 cells were plated into 96-well plates and cultured in phenol red-free DMEM plus 1% charcoal-treated FBS. After culture for 24 h, cells were treated with Tam (100 nM) or compounds (100 nM) for 6 h. And then cells were fixed, permeabilized, and incubated with an ER*α*-antibody (SC-8002, Santa Cruz, dilution 1/200), and Alexa Fluor® 488 anti-mouse antibody [27]. LNCaP cells were cultured on poly-D-lysine-coated cover wells in phenol red-free RPMI 1640 plus 1% cs-FBS overnight and treated with compounds for 1 h. DHT was added to a final concentration of 10 nM. After 1 h, cells were fixed, permeabilized, and incubated with an anti-AR antibody (ab3510, Abcam, dilution 1/1000) and an Alexa Fluor 488 donkey anti-rabbit IgG (H+L) antibody [28, 29]. Nuclei were counterstained with Hoechst 33342. Images were captured at 20x magnification using a PerkinElmer High content screening system.

### 2.5. Transient Transfection and Luciferase Reporter Assay

Hela cells were seeded in 24-well plates at a density to become 70–90% confluent when they are attached. Transient transfection was performed by using the Lipofectamine and plus reagents following the manufacturer's instructions. Cells were cotransfected either with 0.2 *μ*g ER*α* plasmid, 0.4 *μ*g pERE-luc, and 0.2 *μ*g pTk-Renilla or 0.2 *μ*g ER*β* plasmid, 0.4 *μ*g pERE-luc, and 0.2 *μ*g pTk-Renilla per well. After incubating for 6 h, cells were treated with compounds. AD293 cells were plated in 96-well plates in growth medium of 1% CD-FBS without antibiotics at a density to reach 90% to 95% confluence at transfection. After attachment and growth for 24 h, the cells were cotransfected with the reporter plasmid pTk-ARE-Luc and AR. Transfection was carried out for 18 h in serum-free, antibiotic-free RPMI 1640 media using Lipofectamine. Luciferase activity was then assayed after additional 24 h incubation by using the Luciferase Assay System (Promega). The Renilla luciferase activity was used to normalize that of firefly luciferase.

### 2.6. MTT Assay

Cell proliferation was studied by using MTT assay. Cells were seeded (MCF-7 cells, 10^4^/well in 24-well plates; LNCaP cells, 8000/well in 96 wells plate) and, after attachment, cells were treated with various concentrations of compounds in DMSO for 72 h. OD570 values of compounds were detected using a TECAN Infinite® 200 PRO NanoQuant multimode microplate reader.

### 2.7. Animals and Hormonal Manipulations

A total number of 18 Wistar male rats (250–300 g) were obtained from Beijing Vital River Laboratory Animal Technology Co., Ltd. in China. The experiments and animal care were conducted in accordance with the guidelines of the Chinese Council on Animal Care and approved by the Tianjin University of Traditional Chinese Medicine Animal Care and Use Committee.

BPH rat model was conducted as the previous method [30]. In brief, 6 rats were randomly separated into a sham-operated group, and the other 12 rats were castrated and randomly assigned to two experimental groups with 6 rats per group. All rats were maintained in an animal facility under standard laboratory conditions for 3 weeks. The specific experimental treatments on each group were listed in Table 1. The ratio of oestradiol benzoate and testosterone propionate was 1 : 100 (E/T = 10 *μ*g/1000 *μ*g) [30, 31], subcutaneous daily injection of the mixed solutions to the castrated 12 rats. As vehicle, the sham-operated rats were daily subcutaneously injected with 0.1 mL of corn oil. CS extract was orally given for 45 days. Rats were under the chloral hydrate anesthesia and weighed 24 h after the last injection. The whole prostates were dissected and weighed for calculating the prostatic index (PI). One ventral lobe of the prostate was fixed in phosphate-buffered formalin and embedded in paraffin for histological and immunohistochemical studies. And the other ventral lobe was stored at −80°C for the protein and RNA extraction.

### 2.8. Calculation of PI

The formula for calculating the prostatic index (PI) was as follows [30]:

(1) $$\begin{array}{c} \text{PI}=\frac{\text{gross wet weight of prostate}}{\text{body weight of the whole rat}}\times 100\%. \end{array}$$

### 2.9. Histological and Immunohistochemical Studies

Haematoxylin and eosin (H&E) staining and immunohistochemical (IHC) staining were performed as previously described [30]. Briefly, 5 *μ*m sections of one ventral lobe of the prostate were deparaffinized in xylene and rehydrated in a graded series of alcohol. One 5-*μ*m section was stained with haematoxylin and eosin (H&E) for histological examination. Another 5-*μ*m section was using the avidin–biotin–peroxidase complex method to process immunohistochemistry. The endogenous peroxidase activity was blocked with 0.3% hydrogen peroxide at room temperature for 10 min, followed by incubation with 10% serum at 37°C for 1 hour. Sections were incubated with primary antibody at 4°C overnight. The primary antibody was anti-PCNA antibody (proliferating cell nuclear antigen, 1/200, Pro-tech,). Then the sections were added the biotinylated secondary antibody at 37°C for 1 hour, followed by peroxidase-labelled streptavidin. The secondary antibody was biotinylated goat anti-rabbit IgG (1/200, ZSGB-BIO). Finally the sections were stained by the DAB (boster) and hematoxylin, followed by dehydration and transparency in a graded series of alcohol and dimethylbenzene.

### 2.10. Determination of Estradiol and Testosterone Level in Serum

The blood samples of rats were centrifuged at 3,000 rpm for 10 min at room temperature. The supernatant of the blood samples was collected and then stored at ultra-low temperature freezer. The concentrations of estradiol and testosterone in serum were determined by the enzyme-linked immunosorbent assay [30].

### 2.11. Real-Time Quantitative PCR Analysis

Total RNA was extracted from the frozen ventral lobe of the prostate tissue using Trizol reagent (TIANGEN) according to the manufacturer's protocol. Real-time quantitative PCR was carried out with the PCR primers as Table 2. The conditions of Real-time quantitative PCR included preheating at 95°C for 5 min, and then followed by 39 cycles of 95°C for 30 s, 55°C for 30 s, and 72°C for 30 s. The mRNA level of relative gene expression was determined by the comparative CT method and normalized to the housekeeping gene GAPDH.

### 2.12. Western Blot Assay

Protein was extracted from the ventral lobe of prostate for each group, and the concentration was determined according to the manufacturer's instructions (BCA Protein Assay Kit, Thermo Fisher). 40 *μ*g proteins were loaded into the SDS-PAGE. Following gel electrophoresis (SDS-PAGE), gel was transferred onto PVDF membrane (Millipore, Billerica, MA, USA) and incubated in TBST buffer, supplemented with 5% milk as the blocking buffer for 1 h. Next the membrane was incubated with primary antibodies under the 4°C rotating overnight. The primary antibodies were PCNA (10205-Z-AP, Pro-tech, dilution 1/2000), AR (ab3510, Abcam, dilution 1/500), ER*α* (SC-8002, Santa Cruz, dilution 1/500), ER*β* (SC-8974, Santa Cruz, dilution 1/500), and GAPDH (B0004-1-lg, Pro-tech, dilution 1/2000). The PVDF membrane was washed five times with TBST and then incubated with the appropriate secondary antibodies conjugated to horseradish peroxidase and detected next by Enhanced chemiluminescence.

### 2.13. Statistical Analysis

All results were presented as mean ± standard deviation (SD). Statistical significance was determined with One-Way ANOVA. ^*∗*^*P* < 0.05, ^*∗∗*^*P* < 0.01, and ^*∗∗∗*^*P* < 0.001 were considered statistically significant.

## 3. Results

### 3.1. Compounds of CS Extract Showed Phytoestrogenic Activity In Vitro

**Figure 2:**
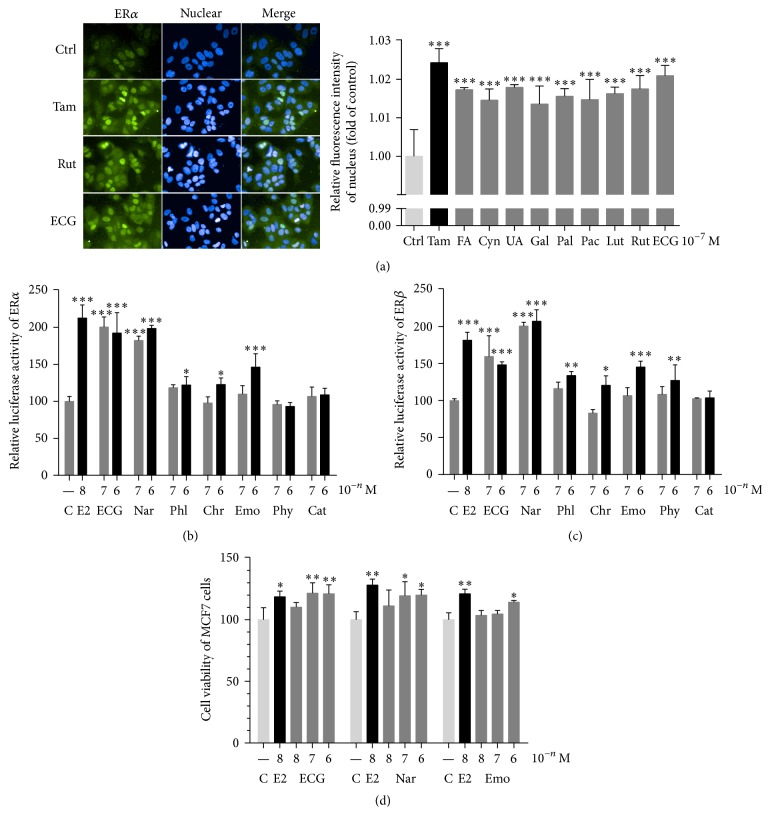
Compounds from CS extract showed phytoestrogenic activity in vitro. (a) Visualization of intracellular ER*α* in MCF-7 cells (left) and quantification of fluorescence intensity after treatment with compounds (right). (b), (c) ER*α* and ER*β* transcriptional activity were activated after treatment with compounds. (d) The proliferation of MCF-7 cells was induced after treatment with compounds. Compare with control, ^*∗*^*P* < 0.05, ^*∗∗*^*P* < 0.01, and ^*∗∗∗*^*P* < 0.001.

In the absence of estrogen, ER*α* was distributed throughout the cell. Following stimulation with Tam, the nuclear staining of ER*α* was increased dramatically. Similarly, when treating with compounds FA, Cyn, UA, Gal, Pal, Pae, Lut, Rut, or ECG, ER*α* locations in the nucleus were also significantly increased in MCF-7 cells (Figure 2(a)), indicating their possible phytoestrogenic activities. Further investigation by dual-luciferase assay showed that ECG and Nar upregulated ER*α* transcriptional activity at 10^−7^ M and 10^−6^ M, while Phl, Chr, and Emo promoted ER*α* transcription activity at 10^−6^ M (Figure 2(b)). These results suggest that compounds of CS extract exhibit estrogenic like activity by facilitating ER*α* translocation to nuclear and activated ER*α* transcriptional activity. Since not only ER*α*, but also ER*β* plays a role in estrogen-stimulated genomic effects, we also detected the ER*β* transcriptional activities after treatment with compounds. ECG and Nar upregulated ER*β* transcriptional activity at 10^−7^ M and 10^−6^ M, and Phl, Chr, Emo, and Phy promoted ER*β* transcription activity at 10^−6^ M (Figure 2(c)). Considering the selectivity of ER*α* and ER*β* and different affinities to ER*α* and ER*β* with different concentrations, we thought that compounds from CS exhibit estrogenic activities depending on different conditions. To further confirm the effect of compounds on estrogenic like function, we did MTT assay. As shown in Figure 2(d), ECG, Nar, and Emo accelerated proliferation of MCF-7 cells as estradiol did.

### 3.2. Compounds of CS Extract Showed Phytoandrogenic Activity In Vitro

**Figure 3:**
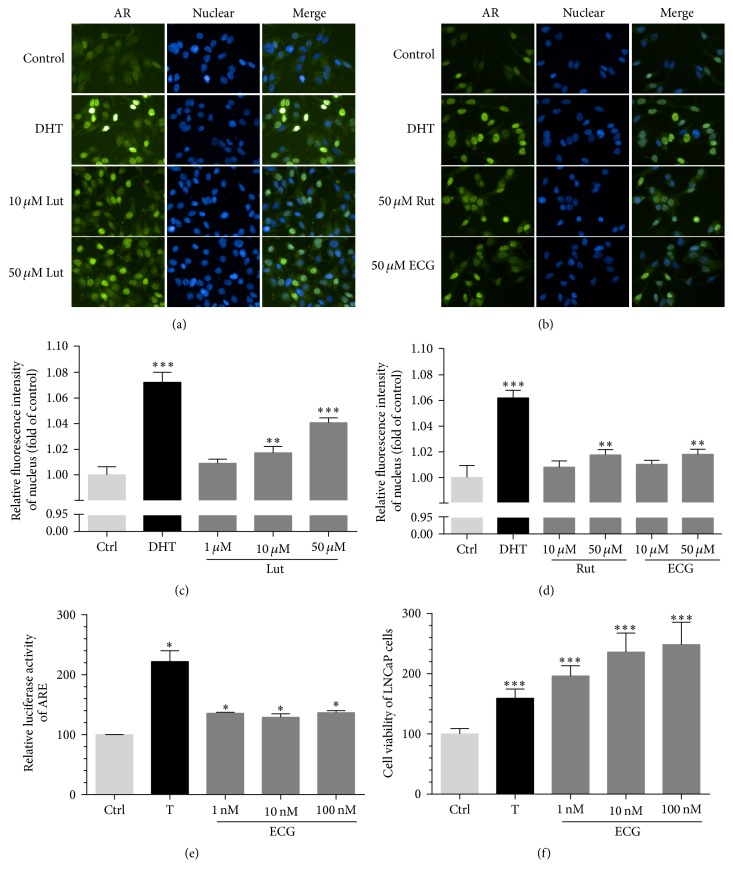
Compounds from CS extract showed phytoandrogenic activities in vitro. (a) Visualization of intracellular AR in LNCaP cells when incubated with DHT or Lut. (b) Visualization of intracellular AR in LNCaP cells when incubated with DHT, Rut, or ECG. (c), (d) The relative fluorescence intensity of AR in nuclear was quantified after treatment with Lut, Rut, or ECG. (e) AR transcriptional activity was activated by ECG in AD293 cells. (f) The proliferation of LNCaP cells was induced by ECG. Compare with control, ^*∗*^*P* < 0.05, ^*∗∗*^*P* < 0.01, and ^*∗∗∗*^*P* < 0.001.

We also investigated the phytoandrogenic activities of compounds from CS. In the absence of androgen, AR was mainly distributed in the cytoplasm of LNCaP cells. After 1 h stimulation with DHT, the nuclear staining of AR was increased obviously. AR locations in the nucleus were also highly increased in a dose-dependent manner when treated with compounds Lut, Rut, and ECG (Figures 3(a), 3(b), 3(c), and 3(d)), while FA, Cyn, UA, Gal, Pal, and Pac have no obvious effects (data not shown). We also found ECG induced ARE luciferase activity and androgen-dependent LNCaP proliferation in a dose-dependent manner as testosterone did (Figures 3(e) and 3(f)), while Lut and Rut have no obvious effects (data not shown).

### 3.3. CS Extract Inhibited the Estrogen-Androgen Induced-BPH Progress In Vivo

**Figure 4:**
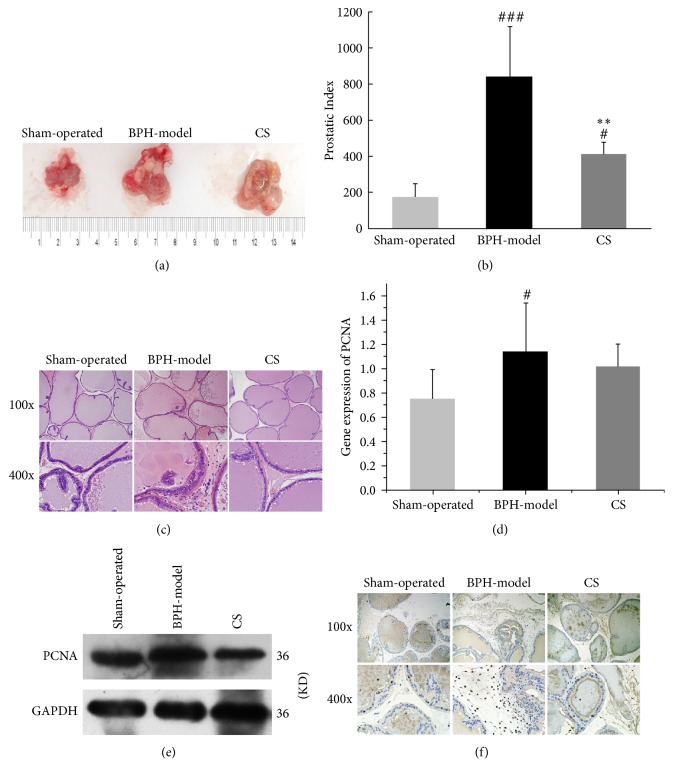
CS extract inhibited the estrogen-androgen induced-BPH progress in vivo. (a) The prostatic morphologies and (b) the prostatic index of rats were shown and calculated in sham-operated group, BPH model, and CS groups. (c) The pathophysiology of rat prostate was analyzed by HE staining in sham-operated group, BPH model, and CS groups. (d) The mRNA and (e) protein expressions and (f) distributions of PCNA in prostate tissues were investigated and quantified by RT-qPCR, western blot, and immunohistochemical staining. Compare with sham-operated group, ^#^*P* < 0.05, ^###^*P* < 0.001; Compare with BPH model, ^*∗∗*^*P* < 0.01.

Compared with those in the sham-operated group, the sizes and weights of the prostate in BPH model group were significantly increased, which was further decreased by CS administration (Figures 4(a) and 4(b)). By pathological analysis, thickness of the periglandular smooth muscle layer and the height of the luminal cells were significantly increased in BPH model group, which was decreased after CS administration (Figure 4(c)). The mRNA and protein expressions and distribution of PCNA in the prostate were also upregulated in the BPH model group comparing with the sham-operated group, which were then suppressed in CS group (Figures 4(d), 4(e), and 4(f)).

### 3.4. Effects of CS Extract on AR and ER

**Figure 5:**
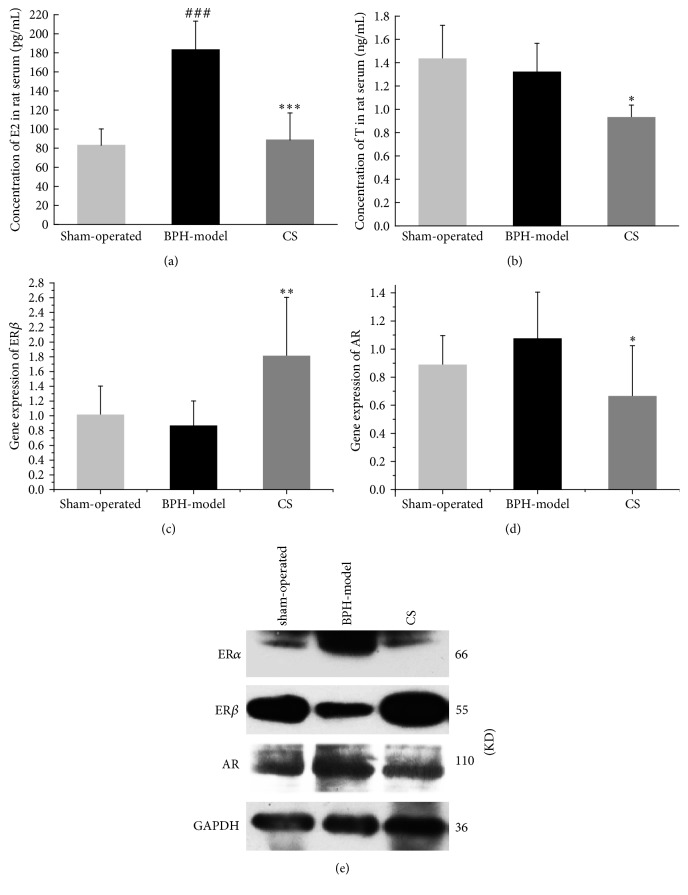
Effects of CS extract on AR and ER in vivo. (a), (b) The levels of estradiol (E2) and testosterone (T) in serum were detected by ELISA in sham-operated group, BPH model, and CS groups. (c), (d) Gene expressions of ER*β* and AR in prostate of rats were tested by RT-qPCR in sham-operated group, BPH model, and CS groups. (e) Protein expressions of ER*α*, ER*β*, and AR were determined by western blot in sham-operated group, BPH model, and CS groups. Compare with sham-operated group, ^###^*P* < 0.001; compare with BPH model, ^*∗*^*P* < 0.05, ^*∗∗*^*P* < 0.01, and ^*∗∗∗*^*P* < 0.001.

Since compounds from CS have phytoestrogenic and phytoandrogenic activities, and estrogen and androgen play important roles in BPH development, we wondered whether the suppression of BPH by CS is mediated by interfering with the estrogen and/or androgen signaling. As shown in Figures 5(a) and 5(b), E2 and T levels in serum CS group were significantly lower than those in BPH model group. ER*α* expression was suppressed at protein level in CS extract treatment group. Besides, ER*β* expressions were increased and AR expressions were decreased in CS extract treatment group compared with BPH model group at levels of both mRNA and protein (Figures 5(c), 5(d), and 5(e)).

## 4. Discussion

Phytoestrogens are a diverse group of natural compounds that have the abilities to act as estrogenic or/and antiestrogenic functions [32]. Studies have shown that lots of traditional medicines produce compounds that may mimic estrogenic effect and thus considered as typical phytoestrogens [7, 33, 34]. In our study, we first showed that several compounds from CS have phytoestrogenic activities (Table 3) by increasing ER*α* translocation to nucleus, inducing ERE luciferase activity, and/or enhancing MCF-7 proliferation (Figure 2), indicating that CS is such kind of traditional medicines that may participate in the estrogen signaling pathway and regulate the abnormal signaling involved in the estrogen-induced diseases. Meanwhile, it is also worth noting that compounds that were used in our distinct assays did not overlap with each other. We found that all of the nine detected compounds (FA, Cyn, UA, Gal, Pal, Pae, Lut, Rut, and ECG) promoted ER*α* nuclear translocation (Figure 2(a)) in the concentration of 100 nM, whereas only two of seven detected compounds (ECG and Nar) significantly induced ER*α* transcriptional activities at 100 nM (Figure 2(b)), which further confirmed their phytoestrogenic effects by proliferation of MCF-7 cells (Figure 2(d)). Our finding indicated that ER translocation to nucleus is the necessary step induced by compounds to mimic estradiol genomic effects, but it is not sufficient. The competitive ability with estradiol by ER recruitment and selective manner of the target genes involved in certain biofunctions such as cell proliferation are also worth noting. Therefore, we cannot exclude that compounds with positive results from one assay may have different effects or may behave negative readouts from other assays. ECG is one of the important compounds from CS, and our results first proved that ECG has strong and consistent results on the phytoestrogenic activity (Figures 2(a), 2(b), 2(c), and 2(d)). Previous study has reported that Lut has estrogenic activity [35]. Here we also found that Lut promoted ER*α* nuclear translocation. On the selectivity of ER subtypes, we did not observe the specific ER*α* or ER*β* selectivity among seven detected compounds (ECG, Nar, Phl, Chr, Emo, Phy, and Cat) (Figures 2(b) and 2(c)).

Similar to phytoestrogens, the concept of phytoandrogen has also been recognized and valued [9]. Traditional medicines that were reported for treatment of syndromes including impotence, infertility, and erectile dysfunction in clinically are a large class of phytoandrogen pool containing hopeful candidates with androgenic activities, while the related reports are still not too much [36]. CS is a well-known and widely used traditional medicine applied to reinforce yang in TCM. Here, we first demonstrated that ECG, Lut, and Rut significantly induced AR translocation to nuclear (Figures 3(a) and 3(b)), and ECG upregulated AR transcriptional activities (Figure 3(e)) and stimulated androgen-dependent LNCaP cell proliferation in a dose-dependent manner (Figure 3(f)), providing evidence that CS contains potential phytoandrogens (Table 3).

Combining the results of estrogenic and androgenic activities, it is interesting and notable to find that ECG is the only one with dual activities of estrogen and androgen among all isolated compounds from CS (Table 3). Previously, ECG has been reported as a naturally occurring polyphenolic compound with putative antioxidant, anti-inflammatory, antibacterial, and antitumor activities [37–41]. Here, we first demonstrated its function involved in hormone related signaling and regulation.

It has been well accepted that both estrogen and androgen play key roles during BPH development [42]. Here, we used estradiol and testosterone cooperatively induced rat BPH model [30, 43, 44] to detect the inhibitory effect of CS in BPH development. As we expected, the prostate volume, PI, and PCNA expression in BPH group were dramatically increased comparing with those in the sham-group, which were further inhibited in CS group (Figures 4(a), 4(b), 4(d), 4(e), and 4(f)), providing evidence that CS is an efficient administration for BPH treatment in accordance with the clinical experience in China. Recently, two publications showed that Lut and UA, two of the compounds isolated from CS, have significant effects in inhibiting prostate-related diseases. Lut inhibited cell proliferation and induced apoptosis in LNCaP human prostate cancer cells by mediated AR downregulation [45]. UA significantly decreased the prostate size, prostatic hyperplasia, and DHT levels in the serum and prostate of BPH rat, strongly suggesting it can be utilized as a useful agent in BPH treatment [46]. However, concerning the phytoestrogenic or/and phytoandrogenic activities of different compounds from CS, mainly compounds that are involved in and contributed to anti-BPH effects of CS still need to be studied in the future.

As we known, ER*α*, ER*β*, and AR are nuclear receptors that mediate estrogen and androgen signaling. In prostatic stromal cells, ER*α* and AR are highly expressed and the increased levels of which are considered to contribute the BPH progression [47, 48], while, in contrast, ER*β* and AR are highly expressed in prostatic epithelial cells; the upregulation of AR and downregulation of ER*β* are related to BPH [49, 50]. In our study, we found the CS group significantly inhibited ER*α* and AR and induced ER*β*, particularly in protein levels. These data suggested that CS exhibits antiestrogen and antiandrogen effects that finally inhibited BPH development.

Selective estrogen receptor modulators (SERMs) and selective androgen receptor modulators (SARMs) are two classes of drugs under development for a variety of diseases due to their high specificity for ER or AR, selective anabolic activity, lack of virilizing side effect, and ability to extend estrogen or androgen therapy [51]. Due to their unique abilities to selectively act as agonists or antagonists in a target gene and in a tissue-specific fashion [52], SERMs and SARMs are now being used as treatment for breast cancer, osteoporosis, postmenopausal symptoms, prostate cancer, and BPH [53–55]. We found that compounds from CS act as agonists of estrogen and/or androgen in HeLa, MCF-7, AD293, or LNCaP cells, while in vivo CS extract acted as antagonists for anti-BPH, indicating the potential role of CS and its active compounds as SERM or SARM.

Taken together, our findings demonstrated that CS inhibits rat BPH via interfering with estrogenic and androgenic signaling, thereby offering the potent candidates from CS as SERMs or SARMs for related diseases in the future.

## Figures and Tables

**Table 1:** The experimental treatments on each group.

Group	E2/T (in coin oil, s.c.)	Drugs
Sham-operated	0.1 mL coin oil	Normal saline
BPH Model	0.1 mL E2/T	Normal saline
CS extract treatment	0.1 mL E2/T	CS extract (6 g/kg/d)

**Table 2:** List of primer sequences.

Primer name	Primer sequence (5′-3′)	Annealing temperature
PCNA Forward	GAGCAACTTGGAATCCCAGAACAGG	60°C
PCNA Reverse	CCAAGCTCCCCACTCGCAGAAAACT	
AR Forward	GCCGGACATGACAACAACCAGCC	60°C
AR Reverse	AGTGAAGGACCGCCAACCCATGG	
ER*α* Forward	GGTCATAACGATTACATGTG	60°C
ER*α* Reverse	TCTGTCCAAGACCAAGTTAG	
ER*β* Forward	GAGGCAGAAAGTAGCCGGAA	53°C
ER*β* Reverse	CGTGAGAAAGAAGCATCAGGA	
GAPDH Forward	ATGATTCTACCCACGGCAAG	53°C
GAPDH Reverse	CTGGAAGATGGTGATGGGTT	

**Table 3:** Summary of results on cellular level.

Number	Abb.	Androgenic activity			Estrogen activity				
		AR N.T.	AR T.A.	LNCaP Prol.	ER*α* N.T.	ER*α* T.A.	ER*β* N.T.	ER*β* T.A.	MCF7 Prol.
(1)	FA	−	−	N.D.	**+**	N.D.	N.D.	N.D.	N.D.
(2)	Cyn	−	−	N.D.	**+**	N.D.	N.D.	N.D.	N.D.
(3)	UA	−	−	N.D.	**+**	N.D.	N.D.	N.D.	N.D.
(4)	Gal	−	−	N.D.	**+**	N.D.	N.D.	N.D.	N.D.
(5)	Pal	−	−	N.D.	**+**	N.D.	N.D.	N.D.	N.D.
(6)	Pac	−	−	N.D.	**+**	N.D.	N.D.	N.D.	N.D.
(7)	Lut	**+**	−	N.D.	**+**	N.D.	N.D.	N.D.	N.D.
(8)	Rut	**+**	−	N.D.	**+**	N.D.	N.D.	N.D.	N.D.
(9)	ECG	**+**	**+**	**+**	**+**	**+**	N.D.	**+**	**+**
(10)	Nar	N.D.	N.D.	N.D.	N.D.	**+**	N.D.	**+**	**+**
(11)	Phl	N.D.	N.D.	N.D.	N.D.	**+**	N.D.	**+**	−
(12)	Chr	N.D.	N.D.	N.D.	N.D.	**+**	N.D.	**+**	−
(13)	Emo	N.D.	N.D.	N.D.	N.D.	**+**	N.D.	**+**	**+**
(14)	Phy	N.D.	N.D.	N.D.	N.D.	−	N.D.	**+**	−
(15)	Cat	N.D.	N.D.	N.D.	N.D.	−	N.D.	−	−

*Note*. “N.T.”: nuclear translocation, “T.A.”: transcriptional activity, “Prol.”: proliferation, “−”: negative, “+”: positive, and “N.D.”: has not been detected.

## References

[1] Liu H.-P., Chang R.-F., Wu Y.-S., Lin W.-Y., Tsai F.-J. (2012). The Yang-tonifying herbal medicine cynomorium songaricum extends lifespan and delays aging in drosophila. *Evidence-Based Complementary and Alternative Medicine*.

[2] Yang W. M., Kim H. Y., Park S. Y., Kim H.-M., Chang M. S., Park S. K. (2010). Cynomorium songaricum induces spermatogenesis with glial cell-derived neurotrophic factor (GDNF) enhancement in rat testes. *Journal of Ethnopharmacology*.

[3] Lee J. S., Oh H. A., Kwon et al. J. Y. (2013). The effects of cynomorium songaricum on the reproductive activity in male golden hamsters. *Development and Reproduction*.

[4] Ma C., Nakamura N., Miyashiro H., Hattori M., Shimotohno K. (1999). Inhibitory effects of constituents from *Cynomorium songaricum* and related triterpene derivatives on HIV-1 protease. *Chemical and Pharmaceutical Bulletin*.

[5] Jin S., Eerdunbayaer, Doi et al. A. (2012). Polyphenolic constituents of cynomorium songaricum rupr. and antibacterial effect of polymeric proanthocyanidin on methicillin-resistant staphylococcus aureus. *Journal of Agricultural and Food Chemistry*.

[6] Dull A. B., George A. A., Goncharova E. I. (2014). Identification of compounds by high-content screening that induce cytoplasmic to nuclear localization of a fluorescent estrogen receptor *α* chimera and exhibit agonist or antagonist activity in vitro. *Journal of Biomolecular Screening*.

[7] Xin D., Wang H., Yang J. (2010). Phytoestrogens from *Psoralea corylifolia* reveal estrogen receptor-subtype selectivity. *Phytomedicine*.

[8] Maximov P. Y., Lee T. M., Craig Jordan V. (2013). The discovery and development of selective estrogen receptor modulators (SERMs) for clinical practice. *Current Clinical Pharmacology*.

[9] Edouard M. J., Miao L., Fan G.-W. (2014). Yang-tonifying traditional Chinese medicinal plants and their potential phytoandrogenic activity. *Chinese Journal of Natural Medicines*.

[10] Chen J.-J., Chang H.-C. (2007). By modulating androgen receptor coactivators, daidzein may act as a phytoandrogen. *Prostate*.

[11] Tian H. Y., Yuan X. F., Jin et al. L. (2014). A bufadienolide derived androgen receptor antagonist with inhibitory activities against prostate cancer cells. *Chemico-Biological Interactions*.

[12] Hessenkemper W., Roediger J., Bartsch S. (2014). A natural androgen receptor antagonist induces cellular senescence in prostate cancer cells. *Molecular Endocrinology*.

[13] Xu D., Lin T.-H., Li S. (2012). Cryptotanshinone suppresses androgen receptor-mediated growth in androgen dependent and castration resistant prostate cancer cells. *Cancer Letters*.

[14] Roell D., Baniahmad A. (2011). The natural compounds atraric acid and N-butylbenzene-sulfonamide as antagonists of the human androgen receptor and inhibitors of prostate cancer cell growth. *Molecular and Cellular Endocrinology*.

[15] Jang S. Y., Jang E. H., Jeong S. Y., Kim J. (2014). Shikonin inhibits the growth of human prostate cancer cells via modulation of the androgen receptor. *International Journal of Oncology*.

[16] Brooke G. N., Gamble S. C., Hough et al. M. A. (2015). Antiandrogens act as selective androgen receptor modulators at the proteome level in prostate cancer cells. *Molecular and Cellular Proteomics*.

[17] Eaton C. L. (2003). Aetiology and pathogenesis of benign prostatic hyperplasia. *Current Opinion in Urology*.

[18] Ho C. K. M., Habib F. K. (2011). Estrogen and androgen signaling in the pathogenesis of BPH. *Nature Reviews Urology*.

[19] Gail S., Prins L. H., Birch L., Yongbing P. (2006). The role of estrogens in normal and abnormal development of the prostate glan. *Annals of the New York Academy of Sciences*.

[20] Lai K.-P., Huang C.-K., Fang L.-Y. (2013). Targeting stromal androgen receptor suppresses prolactin-driven benign prostatic hyperplasia (BPH). *Molecular Endocrinology*.

[21] Zhang Z., Duan L., Du X. (2008). The proliferative effect of estradiol on human prostate stromal cells is mediated through activation of ERK. *Prostate*.

[22] Shao R., Shi J., Liu H. (2014). Epithelial-to-mesenchymal transition and estrogen receptor *α* mediated epithelial dedifferentiation mark the development of benign prostatic hyperplasia. *Prostate*.

[23] Lu T., Lin W.-J., Izumi K. (2012). Targeting androgen receptor to suppress macrophage-induced EMT and benign prostatic hyperplasia (BPH) development. *Molecular Endocrinology*.

[24] McPherson S. J., Ellem S. J., Patchev V., Fritzemeier K. H., Risbridger G. P. (2006). The role of Er*α* and ER*β* in the prostate: insights from genetic models and isoform-selective ligands. *Ernst Schering Foundation Symposium Proceedings*.

[25] Tao R., Miao L., Wang X. N., Wichai N. (2016). Research progress of effects of *Cynomorium songaricum* Rupr. on the benignprostatic hyperplasia treatment. *The Chinese Journal of Clinical Pharmacology*.

[26] Zhang Q.-H., Wang W.-B., Li J. (2012). Simultaneous determination of catechin, epicatechin and epicatechin gallate in rat plasma by LC-ESI-MS/MS for pharmacokinetic studies after oral administration of Cynomorium songaricum extract. *Journal of Chromatography B: Analytical Technologies in the Biomedical and Life Sciences*.

[27] Gao Q.-G., Chan H.-Y., Man C. W.-Y., Wong M.-S. (2014). Differential ER*α*-mediated rapid estrogenic actions of ginsenoside Rg1 and estren in human breast cancer MCF-7 cells. *Journal of Steroid Biochemistry and Molecular Biology*.

[28] Li J., Cao B., Liu X. (2011). Berberine suppresses androgen receptor signaling in prostate cancer. *Molecular Cancer Therapeutics*.

[29] Titus M. A., Tan J.-A., Gregory C. W. (2009). 14-3-3*η* amplifies androgen receptor actions in prostate cancer. *Clinical Cancer Research*.

[30] Zhou Y., Xiao X.-Q., Chen L.-F. (2009). Proliferation and phenotypic changes of stromal cells in response to varying estrogen/androgen levels in castrated rats. *Asian Journal of Andrology*.

[31] Xiao X., Yuan Q., Wang Y. (2006). Establishment of rats prostatic stromal hyperplasia model. *Acta Scientiarum Naturalium Universitatis Nankaiensis*.

[32] Patisaul H. B., Jefferson W. (2010). The pros and cons of phytoestrogens. *Frontiers in Neuroendocrinology*.

[33] Zhang J.-M., Li J., Liu E.-W. (2016). Danshen enhanced the estrogenic effects of Qing E formula in ovariectomized rats. *BMC Complementary and Alternative Medicine*.

[34] Shi C., Zhu X., Wang J., Long D. (2014). Tanshinone IIA promotes non-amyloidogenic processing of amyloid precursor protein in platelets via estrogen receptor signaling to phosphatidylinositol 3-kinase/Akt. *Biomedical Reports*.

[35] Miksicek R. J. (1993). Commonly occurring plant flavonoids have estrogenic activity. *Molecular Pharmacology*.

[36] Ong V. Y. C., Tan B. K. H. (2007). Novel phytoandrogens and lipidic augmenters from *Eucommia ulmoides*. *BMC Complementary and Alternative Medicine*.

[37] Caturla N., Vera-Samper E., Villalain J., Mateo C. R., Micol V. (2003). The relationship between the antioxidant and the antibacterial properties of galloylated catechins and the structure of phospholipid model membranes. *Free Radical Biology and Medicine*.

[38] Kürbitz C., Heise D., Redmer T. (2011). Epicatechin gallate and catechin gallate are superior to epigallocatechin gallate in growth suppression and anti-inflammatory activities in pancreatic tumor cells. *Cancer Science*.

[39] Baek S. J., Kim J. S., Jackson F. R., Eling T. E., McEntee M. F., Lee S. H. (2004). Epicatechin gallate-induced expression of NAG-1 is associated with growth inhibition and apoptosis in colon cancer cells. *Carcinogenesis*.

[40] Shah S., Stapleton P. D., Taylor P. W. (2008). The polyphenol (-)-epicatechin gallate disrupts the secretion of virulence-related proteins by Staphylococcus aureus. *Letters in Applied Microbiology*.

[41] Stevens C. S., Rosado H., Harvey R. J., Taylor P. W. (2015). Epicatechin gallate, a naturally occurring polyphenol, alters the course of infection with *β*-lactam-resistant Staphylococcus aureus in the zebrafish embryo. *Frontiers in Microbiology*.

[42] Nicholson T. M., Ricke W. A. (2011). Androgens and estrogens in benign prostatic hyperplasia: Past, present and future. *Differentiation; Research in Biological Diversity*.

[43] Wang C., Du X., Yang et al. R. (2015). The prevention and treatment effects of tanshinone IIA on oestrogen/androgen-induced benign prostatic hyperplasia in rats. *The Journal of Steroid Biochemistry and Molecular Biology*.

[44] Wang C., Luo F., Zhou Y. (2016). The therapeutic effects of Docosahexaenoic acid on oestrogen/androgen-induced benign prostatic hyperplasia in rats. *Experimental Cell Research*.

[45] Chiu F.-L., Lin J.-K. (2008). Downregulation of androgen receptor expression by luteolin causes inhibition of cell proliferation and induction of apoptosis in human prostate cancer cells and xenografts. *Prostate*.

[46] Shin I. S., Lee M. Y., Jung D. Y., Seo C. S., Ha H. K., Shin H. K. (2012). Ursolic acid reduces prostate size and dihydrotestosterone level in a rat model of benign prostatic hyperplasia. *Food and Chemical Toxicology*.

[47] Ellem S. J., Risbridger G. P. (2009). The dual, opposing roles of estrogen in the prostate. *Annals of the New York Academy of Sciences*.

[48] Wen S., Chang H.-C., Tian J., Shang Z., Niu Y., Chang C. (2015). Stromal androgen receptor roles in the development of normal prostate, benign prostate hyperplasia, and prostate cancer. *American Journal of Pathology*.

[49] Stanbrough M., Leav I., Kwan P. W. L., Bubley G. J., Balk S. P. (2001). Prostatic intraepithelial neoplasia in mice expressing an androgen receptor transgene in prostate epithelium. *Proceedings of the National Academy of Sciences of the United States of America*.

[50] McPherson S. J., Ellem S. J., Simpson E. R., Patchev V., Fritzemeier K.-H., Risbridger G. P. (2007). Essential role for estrogen receptor beta in stromal-epithelial regulation of prostatic hyperplasia. *Endocrinology*.

[51] Clarke B. L., Khosla S. (2009). New selective estrogen and androgen receptor modulators. *Current Opinion in Rheumatology*.

[52] Martinkovich S., Shah D., Planey S. L., Arnott J. A. (2014). Selective estrogen receptor modulators: Tissue specificity and clinical utility. *Clinical Interventions in Aging*.

[53] Atawia R. T., Tadros M. G., Khalifa A. E., Mosli H. A., Abdel-Naim A. B. (2013). Role of the phytoestrogenic, pro-apoptotic and anti-oxidative properties of silymarin in inhibiting experimental benign prostatic hyperplasia in rats. *Toxicology Letters*.

[54] Gupta S., Afaq F., Mukhtar H. (2002). Involvement of nuclear factor-kappa B, Bax and Bcl-2 in induction of cell cycle arrest and apoptosis by apigenin in human prostate carcinoma cells. *Oncogene*.

[55] Ren F., Zhang S., Mitchell S. H., Butler R., Young C. Y. F. (2000). Tea polyphenols down-regulate the expression of the androgen receptor in LNCaP prostate cancer cells. *Oncogene*.

